# RiTE database: a resource database for genus-wide rice genomics and evolutionary biology

**DOI:** 10.1186/s12864-015-1762-3

**Published:** 2015-07-22

**Authors:** Dario Copetti, Jianwei Zhang, Moaine El Baidouri, Dongying Gao, Jun Wang, Elena Barghini, Rosa M. Cossu, Angelina Angelova, Carlos E. Maldonado L., Stefan Roffler, Hajime Ohyanagi, Thomas Wicker, Chuanzhu Fan, Andrea Zuccolo, Mingsheng Chen, Antonio Costa de Oliveira, Bin Han, Robert Henry, Yue-ie Hsing, Nori Kurata, Wen Wang, Scott A. Jackson, Olivier Panaud, Rod A. Wing

**Affiliations:** Arizona Genomics Institute, BIO5 Institute and School of Plant Sciences, University of Arizona, Tucson, AZ 85721 United States; International Rice Research Institute, Genetic Resource Center, Los Baños, Laguna Philippines; Laboratoire Génome et Développement des Plantes and CNRS and Laboratoire Génome et Développements des Plantes, Université de Perpignan Via Domitia, UMR CNRS/UPVD 5096, 66860 Perpignan, France; Center for Applied Genetic Technologies, University of Georgia, Athens, GA 30602 United States; Department of Biological Sciences, Wayne State University, Detroit, MI 48202 United States; Department of Agriculture, Food, and Environment, University of Pisa, 56124 Pisa, Italy; Institute of Life Sciences, Scuola Superiore Sant’Anna, 56127 Pisa, Italy; School of Life Sciences, Heriot-Watt University, Edinburgh, EH14 4AS Scotland; Institute of Plant Biology, University of Zürich, Zollikerstrasse 107, 8008 Zürich, Switzerland; Plant Genetics Laboratory, National Institute of Genetics, Mishima, Shizuoka 411-8540 Japan; State Key Laboratory of Plant Genomics, Institute of Genetics and Developmental Biology Chinese Academy of Sciences, Beijing, 100101 China; Plant Genomics and Breeding Center, Federal University of Pelotas, Pelotas-RS, Brazil; National Center for Gene Research and Institute of Plant Physiology and Ecology, Shanghai Institutes of Biological Sciences, Chinese Academy of Sciences, Beijing, 100029 China; Queensland Alliance for Agriculture and Food Innovation, University of Queensland, Brisbane, QLD 4072 Australia; Institute of Plant and Microbial Biology, Academia Sinica, Nankang, Taipei, 11529 Taiwan; State Key Laboratory of Genetic Resources and Evolution, Kunming Institute of Zoology, Chinese Academy of Sciences and University of Chinese Academy of Sciences, No. 32 Jiaochang Donglu, Kunming, Yunnan 650223 China

**Keywords:** Rice, *Oryza*, Transposable elements, Repeats, Genome, RiTE-db

## Abstract

**Background:**

Comparative evolutionary analysis of whole genomes requires not only accurate annotation of gene space, but also proper annotation of the repetitive fraction which is often the largest component of most if not all genomes larger than 50 kb in size.

**Results:**

Here we present the Rice TE database (RiTE-db) - a genus-wide collection of transposable elements and repeated sequences across 11 diploid species of the genus *Oryza* and the closely-related out-group *Leersia perrieri*. The database consists of more than 170,000 entries divided into three main types: (i) a classified and curated set of publicly-available repeated sequences, (ii) a set of consensus assemblies of highly-repetitive sequences obtained from genome sequencing surveys of 12 species; and (iii) a set of full-length TEs, identified and extracted from 12 whole genome assemblies.

**Conclusions:**

This is the first report of a repeat dataset that spans the majority of repeat variability within an entire genus, and one that includes complete elements as well as unassembled repeats. The database allows sequence browsing, downloading, and similarity searches. Because of the strategy adopted, the RiTE-db opens a new path to unprecedented direct comparative studies that span the entire nuclear repeat content of 15 million years of *Oryza* diversity.

**Electronic supplementary material:**

The online version of this article (doi:10.1186/s12864-015-1762-3) contains supplementary material, which is available to authorized users.

## Background

Since the advent of second generation sequencing (SGS) technologies, the scientific community has been inundated with hundreds of genome assemblies of varying quality. Assembly quality has a significant impact on genome annotation and all subsequent downstream analyses. In addition to the annotation of gene space, a detailed annotation of repeated sequences and transposable elements (TEs) (*e.g.* presence/absence, types, and location) is critical for understanding the biology of a genome, as well as an important tool for the improvement of high-quality genome assemblies. The adoption of exhaustive repeat libraries developed directly from the genome under investigation is important for minimizing false negatives in the annotation. False negatives arise due to repeat/TE sequence diversification across taxa which can hamper the identification of distinctive repeat features (*i.e.* lineage specific sequences) of the species.

The fastest and most common way to annotate the repetitive fraction of a genome assembly is to create repeat libraries derived from a set of previously identified representative repetitive sequences and screen the target genome using homology-based searches. Unfortunately, this method loses power when the target genome is distantly related to the species used to develop the repeat library. The use of repeat libraries of one species to detect repeats from a related species (that diverged *e.g.* 10 million years ago) is highly problematic as it is known that intergenic space is rapidly evolving for the high turnover rates of transposable elements and repetitive sequence. In the last decade, numerous software tools have been developed to identify complete TEs based on structural features [1, 2]. Such tools are useful for the identification of full-length (FL) and low-copy TEs, but are not suited for quantifying high-copy number TEs – especially when investigating SGS assemblies. Conversely, *de novo* methods such as Piler [3] and RepeatModeler (http://www.repeatmasker.org/RepeatModeler.html) have been developed to identify repeats in genome assemblies based on the number of times a sequence motif appears in an input file. Other tools, such as ReAS and RepeatExplorer (RE) [4, 5] apply the same principle but importantly do not rely on a genome assembly to isolate the repeat fraction. Unlike ReAS, RE is designed to use SGS data as input, thereby producing a more granular representation of a genome at lower cost and with higher resolution.

Unfortunately, high-copy number repeats and transposable elements are not well represented in most genome assemblies. This is because most current genome assemblies have been generated using short-read chemistry (*e.g.* Illumina): highly repetitive sequences and transposable elements pose significant problems to assembly algorithms. As a result, repeat annotation of most SGS assemblies leads to skewed representations of the actual native repeat content. Therefore the use of software tools such as ReAS and RE can lead to a more accurate and complete representation of the repeat content of a given genome.

The majority of available repeat data for plants is derived from a few crops and model species. However, the development of specific repeat libraries for new genome projects is now becoming a routine. The dissemination of repeat libraries organized in databases is a common mechanism used to provide informative and convenient access to repeat and TE sequences. Most repeat databases contain repeat libraries from a single to a few genome assembly projects (*e.g.* [6–8], http://maizetedb.org), or can span the whole spectrum of organisms (http://www.girinst.org).

In rice (*Oryza sativa*), for example, efforts to annotate and distribute rice repeats have led to both generic (http://plantrepeats.plantbiology.msu.edu/) and more specialized databases [9]. Rice has been extensively used as a model species to investigate many aspects of plant biology. In 2005 the International Rice Genome Project published what is still considered the highest quality genome assembly of any crop plant (*i.e.* BAC-by-BAC, Sanger sequenced, < 1/10,000 base error rate across most of the genome [10]). Since 2005 the International *Oryza* Map Alignment Project (I*O*MAP) has generated a large set of genomic resources aimed at exploiting the wild relatives of rice for basic research and crop improvement [11, 12]. This work is targeted towards the generation of a total of 11 highly quality genome assemblies that have been annotated for gene and repeat space (I*O*MAP, in preparation).

Here we present the Rice TE database (RiTE-db), a comprehensive repeat dataset developed with sequence data from 11 *Oryza* species and the outgroup *Leersia perrieri*. Our aim is to provide a complete and detailed annotation of TEs and repeated sequences as a resource to the plant and evolutionary biology communities.

## Methods

RiTE-db is set up on a Linux-Apache-MySQL-Perl (LAMP) system. We use JavaScript libraries including jQuery (1.10.2), jQueryUI (1.10.4) and some additional plugins to perform dynamic web services. We provided keyword and sequence-based BLAST search functions. In the current version, RiTE-db contains 265,549 TE and repeat sequence entries from 11 *Oryza* and one outgroup species. Users can retrieve data with auto-search options by selecting preloaded keywords from database type, species, repeat code and element type, and then download the search results for local use. Users can also use the search result to construct a customized database for remote BLAST searches. The 265,549 entries included in the database resulted from different sources, each detailed separately below. The RiTE-db source code is available upon request.

### Plant Repeats Dataset

It is a collection of plant TE and repeated sequences (PReDa) created by merging datasets from several publicly-available sources: TREP (http://wheat.pw.usda.gov/ITMI/Repeats/), Plant Repeat Databases at Michigan State University (http://plantrepeats.plantbiology.msu.edu/), SoyBase [7, 8], submissions to NCBI, individual publications (*e.g.* [13]) and an in-house collection of repeats from several *Oryza* species (Wing et al., unpublished data). To classify all TE superfamilies and repeated sequences, a modification of the classification system proposed by Wicker and colleagues [14] was adopted, and 48 different codes were used (Additional file 1: Table S1). To remove redundancy, but at the same time retain multiple representatives of each family, a clustering criteria of 90 % sequence similarity was set using USEARCH (http://drive5.com/usearch). Because of the large number of sequences coming from a variety of species and obtained from different sources, PReDa sequences were not classified for degree of autonomy and species.

### Repeat explorer libraries

Single-end reads of 11 *Oryza* species and *L. perrieri* were collected from different sources (Table 1). Chloroplast and mitochondrial reads were isolated by aligning the reads to public sequences [GenBank:NC_001320.1, GenBank:NC_008155.1, GenBank:NC_016677.1, GenBank:NC_007886.1] with Bowtie2 [15]. Non-aligned reads were converted to a fasta format and a random subset spanning ~1.5× of the genome was extracted (Table 1) and used as input for RepeatExplorer (RE) [5]. RE output was further processed and characterized as follows: for each cluster, all sequences with less than 5 % AT or GC content were removed and the remaining reads were assembled with Phrap [16]. Assembled sequences were then aligned to NCBI’s non-redundant protein database (May 2012 release, or later versions) to remove sequences containing non-TE coding sequence. Remaining sequences were masked using the most recently available version of PReDa with RepeatMasker (http://repeatmasker.org). The output was used to characterize sequences down to the superfamily level. Each new dataset produced was characterized using all previously developed databases combined into a single repeat library.

**Table 1:** Features and source materials for the 11 *Oryza* species and *Leersia perrieri*. Approximately 1.5× of single-end raw Illumina reads was used for *de novo* repeat library construction and assembled sequences were used to detect full-length elements. Two independent RE libraries were developed for each of the two *O. sativa* subspecies. The genome assembly of *O. officinalis* was not available for the full-length element characterization

Species	Genome Type	Est. Genome Size		Illumina Reads			Assembled Genome		
		Mb	Ref.^a^	Avg. Read Length (bp)	# of Reads (M)	Source	Status^b^	Size(Mb)	Source
*O. sativa.* ssp. *japonica*	AA	389	1	97	6.5	R. Wing – Unpubl.	chr	373.2	http://rice.plantbiology.msu.edu
*O. rufipogon*	AA	439	3	98	6.8	SRA ERR120613	chr	338.0	EMBL-EBI PRJEB4137
*O. sativa.* ssp. *indica*	AA	466	2	100	7.0	R. Wing – Unpubl.	chr	374.5	http://rise2.genomics.org.cn
*O. nivara*	AA	448	3	79	8.7	Y. Hsing – Unpubl.	chr	337.9	NCBI AWHD00000000
*O. glaberrima*	AA	357	4	75	7.2	R. Wing – Unpubl.	chr	285.0	NCBI ADWL00000000
*O. barhii*	AA	411	5	116	5.0	R. Wing – Unpubl.	chr	308.3	NCBI ABRL00000000
*O. glumipatula*	AA	464	5	101	6.9	R. Wing – Unpubl.	chr	372.9	NCBI ALNU02000000
*O. longistaminata*	AA	352	5	75	8.0	W. Wang – Unpubl.	scf	344.6	W. Wang – Unpubl.
*O. meridionalis*	AA	435	5	99	5.9	R. Wing – Unpubl.	chr	335.7	NCBI ALNW00000000
*O. punctata*	BB	425	3	97	6.6	R. Wing – Unpubl.	chr	393.8	NCBI AVCL00000000
*O. officinalis*	CC	651	3	107	9.6	N. Kurata – Unpubl.	-	-	-
*O. brachyantha*	FF	362	3	100	5.5	SRA SRR350707	chr	260.8	NCBI AGAT01000000
*Leersia perrieri*	-	323	6	100	4.9	R. Wing – Unpubl.	chr	266.7	NCBI ALNV00000000

^a^References: a: [10]; b: [29]; c: [30]; d: [31]; e: [32]; f: Arunuganathan K. pers. comm

^b^Status: chr: chromosome pseudomolecules; scf: scaffolds

**Table 2:** Full length TEs. Number of complete elements identified in the 12 genome assemblies. For each element type, both the total and non-redundant amount of complete elements are listed. The redundancy removal and the count of full-length *Helitron* copies were created following a different strategy (see Additional file 2)

Species	LTR-R		TRIM		*SINE*		*MULE*		NA-DNAT	*Helitron*	
	Total	Non-Red.	Total	Non-Red.	Total	Non-Red.	Total	Non-Red.	Total	Tot. Ends	FL copies
*O. sativa.* ssp. *japonica*	3240	1278	2911	11	280	230	10468	172	77	-	-
*O. rufipogon*	1498	945	2724	11	276	197	10504	172	-	-	-
*O. sativa.* ssp. *indica*	1905	1253	3252	11	288	274	9596	168	-	-	-
*O. nivara*	1283	945	2248	11	232	175	10176	168	-	-	-
*O. glaberrima*	1520	865	2297	11	221	106	7416	162	93	-	-
*O. barhii*	1041	580	2384	11	244	206	9863	169	-	-	-
*O. glumipatula*	637	424	2470	11	264	200	9205	169	-	-	-
*O. longistaminata*	77	59	2344	11	344	293	5910	156	-	2279	338
*O. meridionalis*	339	288	2351	11	257	234	7316	162	-	2122	714
*O. punctata*	2906	2359	1659	7	120	103	6720	147	-	2437	949
*O. brachyantha*	395	360	1699	12	13	11	4963	104	-	-	-
*Leersia perrieri*	872	810	1624	6	116	110	3541	115	-	893	196
Total	15713	10166	27963	124	2655	2139	95678	1864	170	7731	2197

### Long terminal repeat retrotransposons

*De novo* prediction of long terminal repeat retrotransposons (LTR-RTs) for each *Oryza* species was performed using LTRharvest software (http://www.zbh.uni-hamburg.de/?id=206; [17]) with the same parameters as in [18]. All candidate sequences composed of tandem repeats were removed using Tandem Repeats Finder [19]. LTR-RTs were masked with PReDa using all non-LTR-RT repeats to avoid the presence of nested insertions. Families of paralogous elements were defined as previously described by Wicker *et al*. [14]. Any family with less than three copies that did not contain any domains related to *Copia* or *Gypsy* elements was removed from the dataset. To reduce the redundancy of highly repeated families while conserving their diversity, centroid sequences were defined using cluster_fast with centroids option (d = 80), as part of the UCLUST package (www.drive5.com/usearch/). A total of 10,166 complete non-redundant LTR-RTs was indentified (Table 2). The same method adopted to define families within a species was applied to all the elements for all 12 genomes, defining families shared between two or more assemblies. Information about single insertions were encoded in the sequence header in the following way: [Wicker code]_#[Order/Superfamily]_[Species]_[Family id1]_[Family Id2]_[Chromosome]_[Start]_[End] [Corresponding elements in Retroryza] [Full length or Fragment]. For example: the LTR-RT copy named RLG_#LTR/Gypsy_Ojapo1_fam7_Chr4_7056110_7070993 hopi FL, is (i) a Gypsy (RLG_#LTR/Gypsy) retro-element (ii) from *O. sativa* ssp. *japonica* (Ojapo), (iii) that belongs to the family number 1 of *O. s.* ssp. *japonica* (this ID can be used to retrieve the other paralogs), (iv) and to family 7 of the whole dataset (to retrieve orthologs). It is located on chromosome 4 (with start and stop coordinates, Chr4_7056110_7070993), (v) shows homology with the Retroryza *hopi* family, and (vi) is a full-length copy (FL).

### Terminal repeat retrotransposons in miniature

TRIMs were annotated by combining *de novo* and homology-based search methods. The 12 genomes were first analyzed with LTR-FINDER software [20] using default options except for minimum LTR size and minimum length between two LTRs that were both decreased to 30 bp. The *O. sativa* tRNA database was used to predict the primer binding site. Output sequences were manually inspected to remove false positives composed of tandem repeats and other non-TRIM sequences. LTR-RTs with homology to reverse transcriptase were removed by BLASTX searches, and only elements shorter than 1.6 kb were considered as TRIMs. Additionally, the identified TRIMs were used to search against other genomes to find homologous TRIMs. Only one representative for each family (for a total of 124 elements, Table 2) was included in RiTE-db.

### Short interspersed nuclear elements

The characterization of full-length short interspersed nuclear elements (*SINEs*) was conducted by adopting the SINE-Finder software tool [13]. Scans for *SINE* elements were performed three times on each genome using different settings and the results were combined. The first run used all default parameters, the second used the target site duplication (TSD) score cutoff set to 7, and the third run had specific A-box and B-box motifs derived from monocotyledonous elements. Outputs were aligned against internal repeat databases and assembled genome sequences to confirm the *SINE* nature and refine the ends. TSDs were removed from each sequence and elements were renamed with a three-letter code [14]. To reduce redundancy, only one copy from two or more 100 % identical elements was retained.

### *MULE* DNA transposons

Terminal inverted repeat (TIR) sequences of Mutator-like elements (*MULE*s) in *Oryza sativa* ssp. *japonica* were obtained from Ferguson et al., [21]. We also scanned for *de novo MULE* TIR sequences in all 12 genomes using RepeatScout (version 1.0.5; [22]). Repeat families with at least 20 copies were collected and consensus sequences were obtained and grouped with known *O. sativa MULE* TIR sequences. To remove sequences from other repeat types, RepeatMasker (http://repeatmasker.org) was run using the classified PReDa sequences as a library. Sequences with at least 30 % of the length masked by known non-*MULE* TEs from PReDa were removed. To examine whether *de novo* consensus sequences represent the TIRs of a *MULE*, RepeatMasker was run on the 12 genome data set with the remaining TIR sequences as the library. Elements that satisfied the following criteria were identified: 1) the element contained two TIR sequences belonging to the same family; 2) the TIRs had opposite orientation; 3) the distance between the two repeats was less than 20 kb; and 4) a 7–11 bp TSD flanks both repeats. Specifically, we allowed a maximum of 2 mismatches/indels in the 9–11 bp TSDs and 1 mismatch/indel in 8 bp TSDs and perfect match in 7 bp TSDs. If a given *de novo* consensus contained at least five members, this sequence was considered to be a *MULE* TIR. Elements that were composed by the two TIR and internal sequence were defined as *MULE* elements. To comprehensively search for *MULE* copies, we used the *de novo*-identified *MULE* TIRs and *O. sativa MULE* TIR to mask the 12 genomes with RepeatMasker. We allowed the TSDs to have a maximum of a 10 bp swing from the putative ends of each element. From the above *MULE* candidates, we further removed the ones containing any known non-*MULE* transposon contained in PReDa (TBLASTN evalue < 1e-9). We further classified elements whose internal sequences were highly similar to annotated non-transposase proteins (TBLASTN evalue < 1e-9) as Pack-*MULE*s. Elements whose sequences were highly similar to *Oryza MULE* transposase proteins (downloaded from NCBI and PReDa) and were longer than 3 kb were classified as autonomous *MULEs*. The main findings are summarized in Additional file 1: Table S4 and described in Supplementary Information.

### Non-autonomous DNA transposons

Candidate non-autonomous DNA transposons (NA-DNATs) sequences were identified by searching each genome assembly in 1000 bp windows (with 200 bp overlaps) for inverted repeats that were flanked by 2 bp (for *Stowaway*) or 3 bp (for *Tourist*) TSD. For *Stowaway*, we searched specifically for the canonical motif CTCCTCCC in the TIR and an adjacent TA target site duplication. For *Tourist*, the only condition was that the TIR began with a G or C and was at least 8 bp long. Approximately 400 candidate sequences were aligned with CLUSTALW [23] to identify groups of sequences that occurred in multiple copies. For each group, a consensus sequence was derived. Redundancy (*e.g.* forward and reverse sequences of the same family) was removed to determine the initial set of families. For each consensus, 30–40 copies were isolated from the genome to produce a second and more robust consensus sequence. Final consensus sequences were then mapped to the genome using BLASTN to identify all copies of each family. In cases where more than one family consensus mapped to the same locus, priority was given to the one that resulted in the longest hit.

### Helitrons

The annotation of these elements was carried out in two steps: identifying a reliable set of *Helitron* ends under stringent conditions, and searching for complete *Helitrons* in the assembled genomes, using data from the previous step under relaxed settings. In the first step, the four genome assemblies were searched for sequences capable of forming hairpin structures using “scan_for_matches” (http://blog.theseed.org/servers/2010/07/scan-for-matches.html) with the pattern: p1 = 7…10 2…4 ~ p1[1,0,0] 6…10 CTRRT. Results were parsed and sequences having low complexity were removed. The remaining sequences were then sorted into putative families, defined by groups of fragments with identical stem sequences. Families having less than 5 elements were not considered. All members of each family underwent a pairwise comparison to identify putative 3’ and 5’ ends: from each candidate a sequence tract encompassing 15 kb upstream and 200 bp downstream from the putative 3’ end was extracted. The tracts were then compared in dot plot analyses using Dotter [24] software, and the results were manually parsed to verify the element’s 3’ end and 5’ ends (Additional file 2: Figure S2). Tracts of 40 bp in length were extracted from the ends of all putative elements and were used to create a *Helitron* ends library. For the second step, the library produced was used to scan all genome assemblies using RepeatMasker (http://repeatmasker.org). Output files were parsed according to the following relaxed criteria: a) 5’ and 3’ ends should have the expected orientation; b) the distance between both ends must be longer than 200 bp and shorter than 15 kb; c) a maximum of 4 mismatches out of 40 bp in each end was permitted; d) no more than 1 mismatch was allowed in the 3’ CTRR end; and e) the *Helitron* AT insertion rule was respected. Any pair of ends fulfilling the above criteria was considered as the ends of a putative complete *Helitron*, for a total of 2197 complete elements (Table 2).

## Results and Discussion

**Fig. 1:**
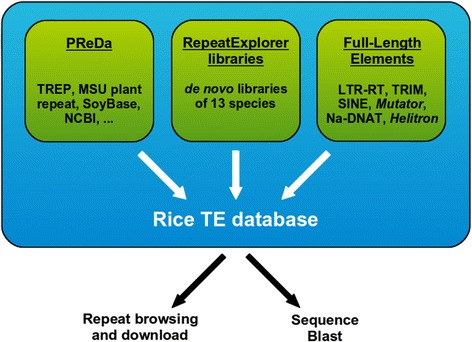
Structure of the Rice TE database. The Rice TE database (RiTE-db) is composed of three data sets: publicly characterized TEs and repeats, *de novo* repeat libraries, and full-length elements isolated from genome assemblies. Result can be downloaded for the users' needs or used to build custom database quires for BLAST searches

RiTE-db contains both published and original data, and is centered on the characterization and public distribution of new repeat sequences from previously uncharacterized and unpublished *Oryza* genome assemblies (Table 1). It is composed of three data sets derived from: 1) the Plant Repeat Database (PReDa), 2) *de novo* repeat assemblies; and 3) full-length (FL) transposable elements (Fig. 1). PReDa is an in house database mostly composed of plant repeats gathered from publicly-available sources. Its role is to represent the current diversity of available plant repeat collections, compensating for the contingent absence of specific repeats from one source, and was used for the characterization of new repeats. *De novo* repeat libraries were developed using RE [5] from raw Illumina data (Table 1), and the output sequences were manually curated and characterized. FL elements were identified using structure-based methods from sequence assemblies of the 11 *Oryza* and the outgroup species (Table 1). Complete insertions were characterized for the following six TE orders: (i) canonical LTR-RTs of the *Copia* and *Gypsy* superfamilies, (ii) terminal repeat retrotransposons in miniature (TRIM), (iii) short interspersed nuclear elements (*SINE*), (iv) *MULE* DNA transposons, (v) non-autonomous DNA transposons (NA-DNAT), and (vi) *Helitrons*. Redundancy was removed from the database by retaining only a single representative of a set of highly similar sequences (*i.e.* > 90 % similarity across the entire length the sequence). All three data sets will be the subject of periodic updates and curation to improve the quality of the sequences.

Sequence classification was based on the published system of Wicker *et al.* [14], with the addition of new codes for other superfamilies and non-TE repeated sequences, such as ribosomal DNA and structural repeats (Additional file 1: Table S1). Of the numerous families of DNA transposons in grass genomes, most are small non-autonomous elements often referred to as miniature inverted-repeat transposable elements (MITEs). However, this term is not useful for systematic classification, because the term MITE includes short transposons from several different superfamilies. Thus we classified NA-DNATs based on conserved sequence motifs in their TIRs and assigned them to corresponding superfamilies. A fourth letter (m) was added to NA-DNAT sequence codes to highlight their non-autonomous nature. The DTX code (that specifies for Class II elements, DNA transposons, uncharacterized superfamily) was assigned to non-autonomous DNA transposons that could not be assigned to a specific superfamily. Similarly, for sequences whose complete classification was not possible, the character X at the corresponding code position was used to denote classification ambiguity. Ribosomal DNA and tRNA sequences were assigned to BRN and BTN codes, respectively. Structural repeats, such as centromeric, telomeric, tandem repeat and low complexity sequences were classified with a code starting with S (Additional file 1: Table S1). Additional file 1: Tables S2 and S3 describe the amount of sequence belonging to different repeat classes in the PReDa and RE datasets, with the individual contribution of each species shown for the latter dataset.

**Fig. 2:**
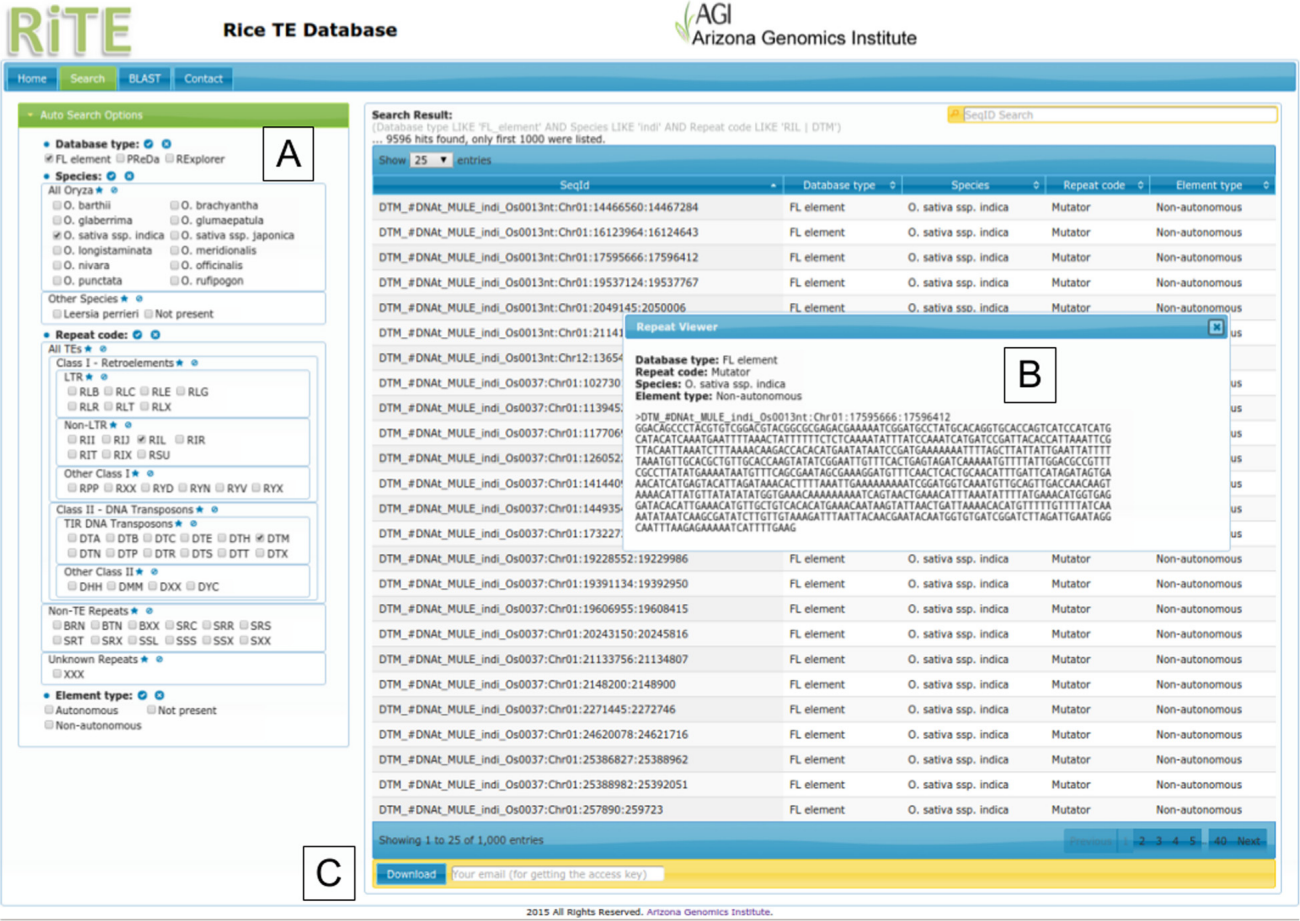
RiTE database search function. Four main classifiers and checkboxes allow for the customization of search parameters (**a**), that are listed and visualized (**b**). Search results can be used as a dynamic BLAST database (**c**) or downloaded locally by using the links provided in via email

**Fig. 3:**
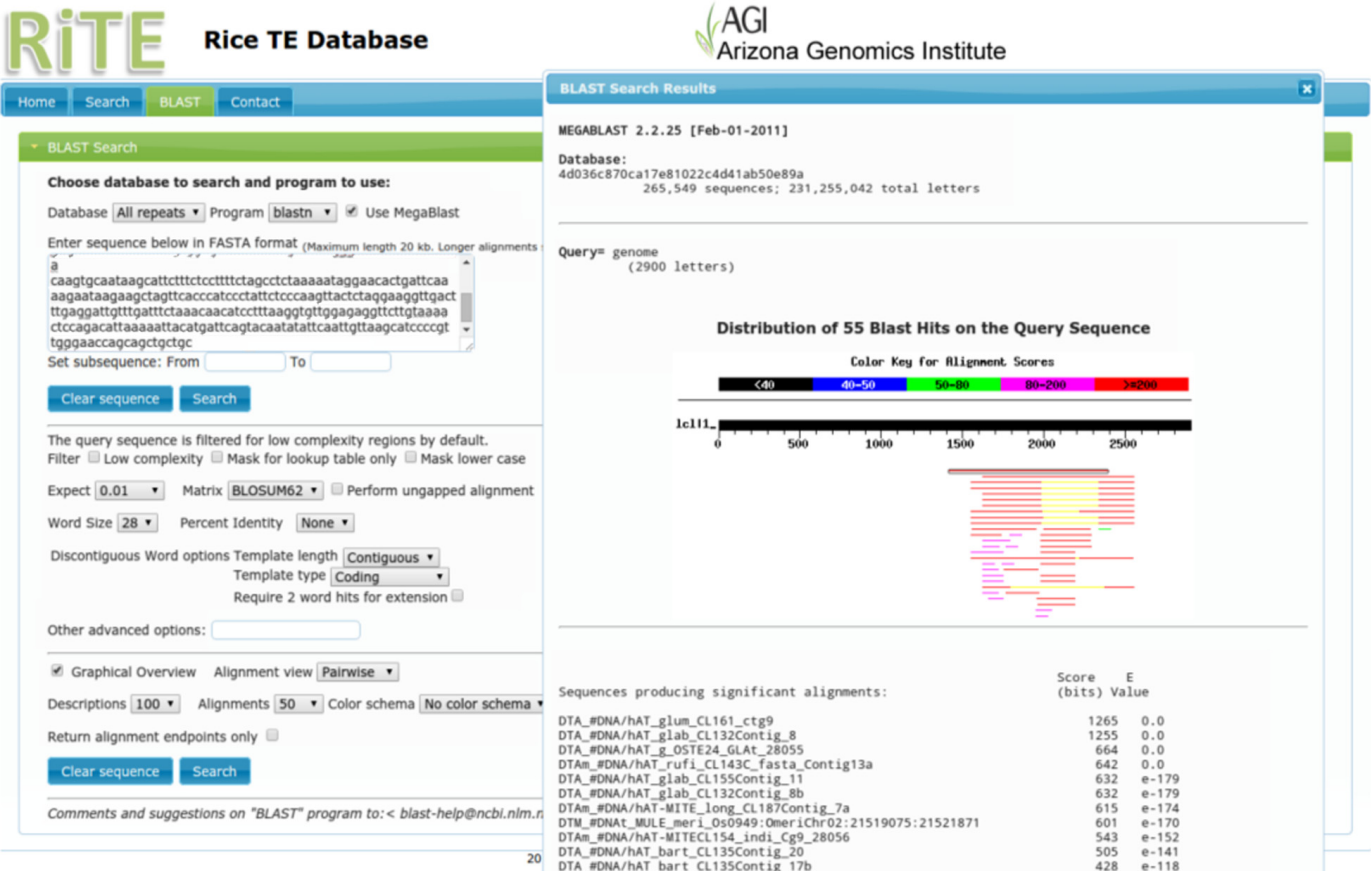
The RiTE database BLAST function. A query sequence can be aligned to the entire RiTE-db or to a customized subset of sequences. Alignment parameters can be tuned and the results can be visualized as pairwise alignments or in a tabular format

The web-based interface developed for RiTE-db allows one to search, browse, visualize, and retrieve sequences (Fig. 2), and perform BLAST searches against them (Fig. 3). Users can conduct searches on the basis of the different criteria used to describe sequence features such as: sequence identifier, element type (from PReDa, RE, or FL), repeat code, and species. The hierarchical structure of sequence classification and species taxonomy features was exploited to retrieve multiple subclasses in a single data base query (*i.e.* selecting Class I, all LTR and non-LTR retrotransposons will be retrieved). A set of checkboxes for each category allows for easy selection of one or more features to conduct searches (Fig. 2a). Selected entries can be then be visualized (Fig. 2b), downloaded (Fig. 2c), or used as a dynamic BLAST database for the custom queries (Fig. 3).

## Conclusions

Rice is the most important food crop in the world. The development of higher yielding and more nutritious and sustainable varieties will play a key food security role as the world population increases to 10 billion in less than 35 years. To help meet this need, the International *Oryza* Map Alignment Project (I*O*MAP) developed a large array of genomic resources that can be used to interrogate the genus *Oryza* (*i.e.* 23 species, 11 genome types, 3.6-fold genome size variation, world-wide distribution, 17 MY of evolutionary divergence) for the identification and harnessing of agriculturally important genes and variation not currently present in modern rice varieties (reviewed in [25]). These resources include a new set of full genome assemblies from eight *Oryza* species ([11, 12] and I*O*MAP *et al*. unpublished), and a single outgroup species *Leersia perrieri*, bringing the total number of full *Oryza* genome sequences to 11. In order to understand and compare the functional and evolutionary diversity of this novel 12 genome data set, our consortia used two genome annotation pipelines for both gene and repeat space annotation, the latter of which is the subject of the development of the Rice TE database (RiTE-db).

The added value of a custom repeat collection comes from the isolation of target sequences by exploiting all of their fundamental features – *i.e.* existing in a high copy number, possessing typical sequence motifs, and/or encoding for specific domains. The *de novo* and assembly-independent approach guaranteed isolation of all repeated sequences regardless of their inclusion in the assembly. The complete elements – displaying specific signatures and isolated from the genome assemblies – enabled the identification of recently transposed or divergent elements, including those present at a single copy. The combination of approaches adopted provided a systematic and uniform survey of repeats across the *Oryza*, allowing for direct comparison across species. Extensive efforts were put in cross-validating all datasets by manual curation to minimize the mis-classification of individual entries or the presence of any other type of sequence different than the one described in the entry (nested TEs, captured gene fragments). Because of how it was conceived, this dataset has its optimal return on genomics research conducted within *Oryza* species. For the investigation of the repeat complement in species outside of this genus, we suggest to use the present method as a template for the production of new and more customized datasets. Given the general conservation of sequence features across plant TEs, most of the methods adopted here can be applied to other plant species with minor modifications. More details are available in the Supplementary Information.

We developed three complementary libraries to which we applied a widely adopted method to classify, handle, and use the data. Distinctions of RiTE-db include that it does not reduce all repeats to a consensus sequence and it spans the breadth of a whole genus. The specificity of the three letter-based classification system adopted [14] allows the user to recognize precisely and associate immediately any transposable element or repeated sequence to a corresponding superfamily. In this study, we expanded the Wicker *et al*. system to incorporate a new set of codes for non-TE repeats and non-autonomous DNATs. This allowed for the incorporation of new types of repeats into the database and to encode information about element transposition capacity.

With this database, we expand the amount of repeat data currently available for the *Oryza* genus in a more curated and uniform manner. For example, RepBase, the most common and complete repeat collection of repetitive sequences (http://www.girinst.org), does not encompass this perspective. Out of 580 RepBase entries (v. 19.04) belonging to the *Oryza* genus, only nine come from species other than *O. sativa* (*i.e.* four from *O. australiensis*, two from *O. glaberrima*, two from *O. longistaminata*, and one from *O. rufipogon*). Besides re-annotating the four published genomes (*i.e. O. sativa* ssp. *japonica* and *indica*, *O. glaberrima*, and *O. brachyantha*), our RiTE-db includes tens of thousands TEs and repetitive sequences for nine new species, where each entry is characterized and classified. The presence of full-length elements is another important step towards building a complete catalog of TEs in a genus. The availability and free circulation of RiTE-db will enable the community to keep pace with the continuous generation of genomic data [26, 27], providing high-quality annotation of any new *Oryza* genome. The breadth and comprehensiveness of RiTE-db was deliberately conceived to help develop Hidden Markov Models of repeated sequences, since scanning genomes with these profiles allows for the identification more ancient or diverged repetitive sequences [28].

We present the Rice TE database (a.k.a. RiTE-db), the first example of a repeat database spanning the breadth of an entire biological genus. The methods used to identify and catalog *Oryza* repeats have been applied consistently across all 12 species, making these datasets directly comparable. The complementarity of the strategies adopted – the detection of all highly repeated sequences and all complete TEs – guarantees the comprehensiveness required for high quality genome annotation. In the future, we envisage the addition of more complete elements from other superfamilies, covering all major plant TE classes. These contributions will support the effort to unlock mechanisms of genome evolution – such as structural or regulatory variations – between species, towards the ultimate goal of linking genetic variation to functional biology and crop improvement.

### Availability

The Rice TE database is freely accessible at www.genome.arizona.edu/rite.

## Additional files

Additional file 1: Table S1–S4. ᅟ Additional file 2: Figure S1–S2. ᅟ
