# Supplementary material for: RiTE database: a resource database for genus-wide rice genomics and evolutionary biology
Source: BMC Genomics. 2015 Jul 22;16(1):538. doi: 10.1186/s12864-015-1762-3 (PMC4508813; doi:10.1186/s12864-015-1762-3)
Supplement: Additional file 2: Figure S1–S2. — ᅟ [file 12864_2015_1762_MOESM2_ESM.doc]

Supplementary Figure 1. Distribution of the fifteen TRIM families identified in 11 *Oryza* species and *Leersia perrieri*. Filled rectangles indicate complete TRIMs, empty rectangles represent partial elements.


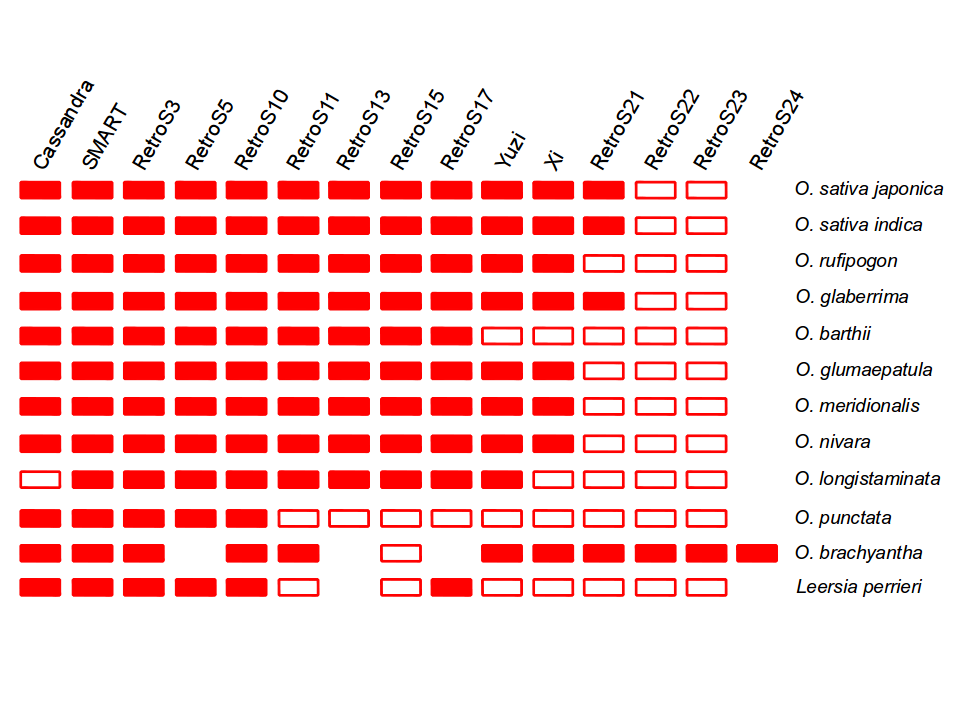


Supplementary Figure 2. Identification of a reliable set of *Helitron* ends. A: Genome assemblies (black lines) were searched for sequences capable of forming hairpin structures and containing 3' consensus sequences (colored boxes). These were considered as *Helitron* putative 3' ends. B: Tracts encompassing 15 kb upstream and 200 bp downstream from the putative 3' end were extracted (lines are not to scale). Stem sequences having low complexity were removed. Remaining tracts were arranged in putative families, on the basis of stem sequences similarity. C: All the members of each family underwent a dot plot comparison to confirm putative 3' ends (and identify 5' ones). If (D) the dot plot shows similarity terminating at proper *Helitron* ends, the element ends are confirmed as true; if (E) similarity extends beyond that point (the CTRRT motif), the putative ends were discarded.


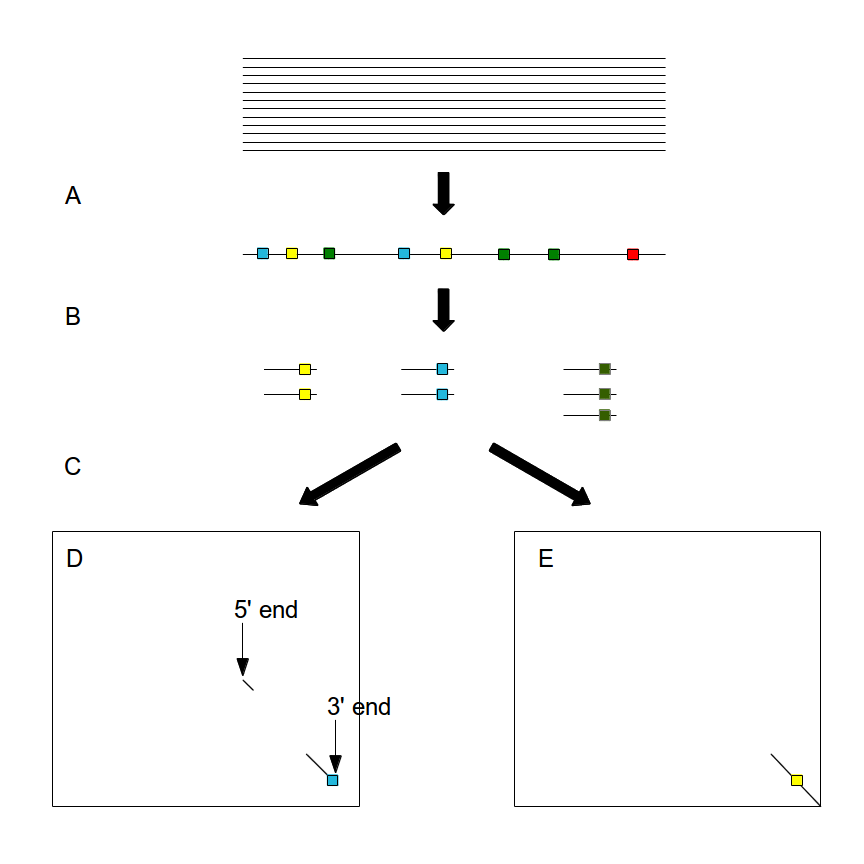


Supplementary Table 1. Codes used to classify repeated sequences and TEs. Several new codes were introduced to the system proposed by Wicker et al. (12). Codes containing X denote ambiguous or unknown classification at the specific level.
